# Serological Hendra Virus Diagnostics Using an Indirect ELISA-Based DIVA Approach with Recombinant Hendra G and N Proteins

**DOI:** 10.3390/microorganisms10061095

**Published:** 2022-05-25

**Authors:** Anne Balkema-Buschmann, Kerstin Fischer, Leanne McNabb, Sandra Diederich, Nagendrakumar Balasubramanian Singanallur, Ute Ziegler, Günther M. Keil, Peter D. Kirkland, Maren Penning, Balal Sadeghi, Glenn Marsh, Jennifer Barr, Axel Colling

**Affiliations:** 1 Institute of Novel and Emerging Infectious Diseases, Friedrich-Loeffler-Institut, 17493 Greifswald-Insel Riems, Germany; kerstin.fischer@fli.de (K.F.); sandra.diederich@fli.de (S.D.); ute.ziegler@fli.de (U.Z.); maren_penning@gmx.net (M.P.); balal.sadeghi@fli.de (B.S.); 2 Australian Centre for Disease Preparedness, CSIRO, Geelong, VIC 3220, Australia; leanne.mcnabb@csiro.au (L.M.); nagendra.singanallur@csiro.au (N.B.S.); glenn.marsh@csiro.au (G.M.); jennifer.barr@csiro.au (J.B.); axel.colling@csiro.au (A.C.); 3 OIE Collaborating Centre for Diagnostic Test Validation Science, Commonwealth Scientific and Industrial Research Organisation, Geelong, VIC 3220, Australia; 4 Institute of Molecular Virology and Cell Biology, Friedrich-Loeffler-Institut, 17493 Greifswald-Insel Riems, Germany; guenther.keil@fli.de; 5 Elizabeth Macarthur Agriculture Institute, Menangle, NSW 2567, Australia; peter.kirkland@dpi.nsw.gov.au

**Keywords:** Hendra virus, horse, ELISA, vaccination, infection, DIVA

## Abstract

Since the identification of Hendra virus (HeV) infections in horses in Australia in 1994, more than 80 outbreaks in horses have been reported, and four out of seven spillover infections in humans had a fatal outcome. With the availability of a subunit vaccine based on the HeV-Glycoprotein (HeV-G), there is a need to serologically **D**ifferentiate the **I**nfected from the **V**accinated **A**nimals (DIVA). We developed an indirect ELISA using HeV-G expressed in *Leishmania tarentolae* and HeV-Nucleoprotein (HeV-N) expressed in recombinant baculovirus-infected insect cells as antigens. During evaluation, we tested panels of sera from naïve, vaccinated and infected horses that either originated from a Hendra-virus free region, or had been pre-tested in validated diagnostic tests. Our data confirm the reliability of this approach, as HeV-N-specific antibodies were only detected in sera from infected horses, while HeV-G-specific antibodies were detected in infected and vaccinated horses with a high level of specificity and sensitivity. Given the excellent correlation of data obtained for German and Australian HeV-negative horses, we assume that this test can be applied for the testing of horse serum samples from a variety of geographical regions.

## 1. Introduction

Henipaviruses belong to the family of *Paramyxoviridae* in the order *Mononegavirales*, carrying an enveloped, single-stranded, non-segmented, negative-sense RNA genome. Hendra virus (HeV) first emerged in 1994 causing respiratory and neurodegenerative disease in horses and humans in Australia [1], and only a few years later, Nipah virus (NiV) was reported to cause comparable symptoms in pigs and humans in Malaysia and Singapore [2]. HeV and NiV, together with the non-pathogenic Cedar virus that was discovered in fruit bats in Australia [3]; Ghana virus, detected in fruit bats in a number of African countries, [4,5]; and the potentially highly pathogenic Moijang virus detected in China [6], form the genus *Henipavirus*. Except for the rodent-associated Moijang virus, pteropid bats have been identified as the natural reservoir of all other henipaviruses [3,5,7,8,9]. All four Australian flying fox species have been reported to harbor HeV-specific antibodies at prevalences of up to 25% [10]. Henipavirus infections appear to be mostly asymptomatic in bats, generally associated with low levels of virus shedding, which may depend on several factors, such as the bat’s reproductive and socioecological status, as well as climatic conditions [11,12,13].

After the report of the first HeV infection in horses and humans in 1994 [1], 26 additional outbreaks were recorded alone in 2011 and 2012 in Queensland and New South Wales. To date, more than 80 outbreaks in horses and seven spillover cases in humans with four fatalities have been reported [14]. In 2012, a recombinant subunit vaccine for horses (Equivac^®^ HeV, Zoetis, Rhodes, NSW, Australia), based on recombinantly expressed HeV-G, was developed [15], and has since been approved by the Australian Pesticides and Veterinary Medicines Authority (APVMA) for use in Australia. However, the vaccination rate in Australian horses has been estimated to remain below 20% [16], leaving unvaccinated horses at risk of an HeV infection, which results in a continuous threat of HeV transmission to humans. This is underlined by continuing reports of HeV cases in horses in the endemic areas.

Diagnostic assays for the detection of Henipavirus infections have been considerably improved since the initial emergence of HeV and NiV infections. At first, the viruses were isolated and virus neutralization tests (VNT) were applied [17], which required handling of live viruses and therefore needed to be performed in a facility of the highest biosafety level 4 (BSL4). Furthermore, these assays were time-consuming as they took at least three days to be completed. Detection of viral RNA by real time RT-PCR is highly sensitive [17,18,19], and is the primary diagnostic tool for confirmation of acute clinical cases by detection of virus in blood and swabs from mucosal surfaces. However, serology is required to identify convalescent horses or horses that may have experienced a subclinical infection due to concerns that humans may have been exposed to infection or that perhaps the infection may recrudesce. International movement of horses from regions where HeV has been detected may also require a negative serology result. The first serological assays depended on the use of HeV-infected cells, cell lysates, or inactivated virus preparations as antigen, again requiring working in a BSL4 facility [17]. Later, recombinant HeV proteins, i.e., the nucleoprotein N and the phosphoprotein P were expressed in E. coli expression systems and used as antigens in indirect ELISA assays [17,20]. These assays displayed a high sensitivity; however, they suffered from a relatively low specificity, resulting in the need to confirm numerous questionable results by VNT [20,21]. Then, an indirect ELISA based on soluble HeV-G (sHeV-G) was developed that showed a distinctly increased sensitivity and specificity [22]. Another monoclonal antibody-based blocking ELISA allowed the specific detection of neutralizing antibodies [23], and recently, a HeV-N-based capture ELISA protocol has been published that allows the early detection of IgM antibodies [24]. Yet another approach is based on microsphere-bound sHeV-G and soluble NiV glycoprotein (sNiV-G) antigens, offering the benefit of being able to use a multiplex assay to differentiate between HeV and NiV infections in one assay [25]. This assay has been widely used for the screening of different animal species for Henipavirus infections [26,27,28]. A direct comparison of the original microsphere-based assay and the indirect ELISA based on sHeV-G protein proved an increased specificity of the microsphere-based assay [29]. However, not all laboratories have access to an expensive reader required for analyzing the microsphere-based assays, while almost all labs are equipped with an ELISA plate reader, so there is still the need for the development and validation of assays that would differentiate between infected and vaccinated horses (DIVA ELISA assays).

We therefore expressed the recombinant HeV-G protein in the protozoan host *Leishmania tarentolae* 30 and the HeV-N protein in Sf9 insect cells using a recombinant baculovirus expression system. Based on these recombinant viral proteins, we developed a DIVA ELISA and validated it for the purpose of differentiating antibodies due to vaccination and infection in comparison with other established diagnostic assays.

## 2. Materials and Methods

### 2.1. Serum Samples

Sera from horses were sourced from the Australian Centre for Disease Preparedness (ACDP) repository based on their disease status (HeV-positive horses; *n* = 21 and Australian negative horses; *n* = 105). Post-vaccination sera were obtained from horses vaccinated with a Hendra virus vaccine containing soluble G (sG; Equivac^®^, Zoetis, Rhodes, NSW, Australia; *n* = 40). German negative horse sera that had been submitted for diagnostic purposes to the National reference laboratory for West Nile Virus were also included (German negative horses; *n* = 288). All sera were heat-inactivated for 30 min at 56°C before use in the assays. Detailed information is provided in Table 1.

### 2.2. Expression of Viral Proteins

HeV-G protein was expressed in *Leishmania tarentolae* as described earlier [30]. Briefly, a sequence coding for the HeV-G protein lacking the N-terminal cytoplasmic tail and the transmembrane domain but harboring an N-terminally fused double Strep-tag coding sequence (iba GmbH, Göttingen, Germany) was codon-optimized for the codon bias of *L. tarentolae*, synthesized and subsequently cloned into the vector pLEXSY-sat2 (Jena Bioscience, Jena, Germany). *L. tarentolae* cells (strain P10, Jena Bioscience) were transfected with the plasmid by electroporation. For selection of positive clones, nourseothricin was used as a selection antibiotic. Since the protein was not secreted into the medium, the recombinant protein was purified from *L. tarentolae* cell lysates using *Strep*-Tactin^®^ Sepharose^®^ (iba GmbH) as described before [30].

HeV-N protein used for the ELISA was expressed in *Spodoptera frugiperda* (Sf9) insect cells (FLI Collection of Cell Lines in Veterinary Medicine (CCLV)) infected with a recombinant baculovirus coding for the HeV-N protein carrying an N-terminal histidine tag. The HeV-N sequence was amplified from HeV RNA (kindly provided by Hana Weingartl, accession number ASB21196) using primers HeV-N fw taacccgggccaccatgagtgatatatttgacgag and HeV-N-rev His-taagcatgcctaatggtgatggtgatggtggctgccgcgagaggccacgtctgctctaacaaagtc and cloned into vector pFDB10UHIS-ieGFPdMCS (derivate of pFast Bac Dual, Life Technologies; modifications available upon request), using restriction enzymes SphI and SmaI. After confirmation of positive pFDB10UHIS-ieGFPdMCS-/Hendra N clones by sequencing, the construct was transformed into DH10Bac competent *E. coli* to generate so-called baculovirus bacmids coding for both HeV-N and GFP. Then, isolated recombinant bacmid DNA was transfected into HighV insect cells using Fugene and incubated for 3 days at 27 °C. Supernatant was serially titrated on Sf9 cells and an agarose-overlay added. After 3 days of incubation at 27 °C, cells were analyzed using a fluorescent microscope, and at least three plaques showing green fluorescence were picked and transferred to fresh medium, establishing the P0 generation of HeV-N-coding baculovirus. This P0 baculovirus generation was then used to inoculate Sf9 cells to produce a P1 generation. Then, 5 to 7 days later, supernatants of infected Sf9 cells were harvested and virus titer was determined by plaque assay. For production of virus stocks, Sf9 cells were infected using an MOI of 0.1 and supernatant was harvested after 6 days and stored at −80 °C until further use. For the preparation of HeV-N, baculovirus-infected Sf9 cells were harvested and centrifuged for 5 min at 1000× *g*. After two washes with phosphate buffered saline (PBS), cell pellets were resuspended in lysis buffer (50 mM NaH2PO4, 300 mM NaCl, 10 mM imidazole, pH 8.0 with EDTA-free protease inhibitor cocktail “cOmplete”, Sigma-Aldrich, Taufkirchen, Germany) and then incubated on ice for 30 min. After ultrasonic homogenization (sonication duration is 65 s at an ultrasonic cycle mode of 15 s sonication and 10 s resting time), lysates were clarified at 20,000 × *g* for 45 min at 4 °C and supernatant was retained for protein purification. HeV-N was purified by incubating supernatant with TALON Metal Affinity Resin (Clontech, Saint-Germain-en-Laye, France) for 2 h at 4 °C. Then, protein/resin slurry was pelleted and washed at least three times with wash buffer (50 mM NaH2PO4, 300 mM NaCl, 20 mM imidazole, 10% glycerin, pH 8.0) before elution of the protein was performed using elution buffer (50 mM NaH2PO4, 300 mM NaCl, 250 mM imidazole, pH 8.0).

### 2.3. Generation of a Rabbit Polyclonal Antibody Raised against HeV-N

A polyclonal antiserum against HeV-N was produced in a rabbit by four consecutive immunizations three weeks apart using 150 µg of HeV-N in elution buffer mixed in a 1:1 ratio with TitermaxGold adjuvant (Sigma-Aldrich). The total volume of 0.4 mL was injected subcutaneously at two different sites. A re-vaccination serum and continuing post-vaccination sera were collected. The final serum was collected three weeks after the final immunization, and is referred to as HeV-N 446.

### 2.4. Development of an Indirect ELISA (FLI HeV DIVA ELISA)

The 96-well MaxiSorp plates (Nunc, Roskilde, Denmark) were coated overnight at 4 °C with 100 ng/well of either HeV-G or HeV-N in 100 µL volume in PBS. During the evaluation process, an equal number of wells were coated in parallel with a cell lysate of untransfected *L. tarentolae* cells to monitor background signal caused by non-specific reactions of the test sera with protozoan proteins. All samples and controls were tested in duplicate. Plates were washed once with PBS containing 0.05% Tween 20 (PBS-T) prior to incubation with 100 µL 5% skim milk in PBS per well for 30 min at 37 °C. Next, the plates were washed with PBS-T before adding 100 µL of the test sera diluted 1/100 in 2.5% skim milk in PBS-T into the respective wells. A no-serum control (NSC), as well as ɣ-irradiated serum from a HeV-infected and a negative horse serum served as positive and negative controls. After incubation at 37 °C for 1 h, plates were washed three times with PBS-T before adding 100 µL of the peroxidase-coupled horse-specific conjugate (goat anti-horse IgG; Dianova, Hamburg, Germany) at a dilution of 1/30,000. The plates were again incubated at 37 °C for 1 h before washing three times with PBS-T. Finally, 100 µL of a TMB solution (Biorad, Feldkirchen, Germany) was added to each well and incubated for 10 min at room temperature (RT) before the reaction was stopped by adding 100 µL of a 1 M H_2_SO_4_ solution to each well. The colorimetric change (optical density; OD) was measured at 450 nm. The OD values were converted to percent positive index values (PP values) using the formula: PP value = mean of test sample/mean of positive control.

### 2.5. Evaluation of HeV-N and HeV-G Assays Using Frequentist Approach and Determination of the Provisional Cut-Off Value

The provisional cut-off was determined as the PP value plus three standard deviations (SD) using 288 HeV-negative German horse sera [31] (Table 1). ROC curve analysis was carried out using sera from 21 HeV-infected Australian horses (Table 1) and 288 HeV-negative German horses to obtain the cut-off for classifying positive and negative results for HeV-G and HeV-N assays using MedCalc^®^ (MedCalc Software, Ostend, Belgium).

### 2.6. Confirmation of ELISA Positive Results

To confirm the ELISA results of selected horse serum samples, we performed Western Blot and immune florescence assays.

### 2.7. Western Blot

Sera of the negative German population with ELISA results above the cut-off value were first re-tested by Western blot analysis. To avoid using the identical antigen as in the ELISA, we used either 1 µg of a commercially available HeV-G protein (Hoelzel Diagnostica, Cologne, Germany) or cell lysates of HEK 293T cells (FLI collection of cell lines in veterinary medicine (CCLV)) transfected with a plasmid coding for the HeV-N protein that were harvested in buffer containing 4% mercaptoethanol at 24 h after transfection. These proteins were separated by sodium dodecylsulfate polyacrylamide gel electrophoresis (SDS-PAGE) and transferred to a PVDF membrane using standard methods. After blocking in 5% skim milk, 1/100 dilutions of the reactive samples were added to the membrane and incubated at RT for 1 h. After washing three times for 10 min with PBS-T, mouse anti-horse peroxidase-coupled conjugate (Dianova, Germany) was added in a dilution of 1/30,000. After three 10 min washing steps in PBS-T, ECL substrate was added to the membrane and the signals were visualized in a VersaDoc Digital Image System (BioRad, Munich, Germany) using the Quantity One and Image software (version 2.3.0.07, BioRad, Munich, Germany). For the determination of the diagnostic sensitivity of this assay, a serial dilution of the positive control serum diluted 1/100–1/25,600 was also applied.

### 2.8. Immunofluorescence Analysis (IF Analysis)

IF analysis was performed as described previously [32] with some modifications. Briefly, Vero76 cells (FLI-CCLV) were transfected with 1 µg plasmid DNA coding for the HeV-N protein. After 24 h, cell monolayers were fixed with 4% paraformaldehyde and permeabilized with 0.2% Triton-X100 (Sigma-Aldrich) in PBS. Sera were incubated in a dilution of 1/100, followed by the incubation with FITC-conjugated secondary anti-horse IgG antibody (1/100 in 5% BSA in PBS). The polyclonal rabbit serum HeV-N 446 in a dilution of 1/200 was used as a positive control and was incubated with a 1/500 dilution of a goat anti-rabbit-Alexa Flour 488 conjugate. Fluorescence was visualized using a DMI7 live cell microscope (Leica, Wetzlar, Germany).

### 2.9. Virus Neutralization Test (VNT)

One serum sample of the negative German population that was reactive in the ELISA and in the Western blot was also tested by VNT. Serial two-fold dilutions of sera were prepared in duplicate in 96-well tissue culture plates in 50 µL cell media (minimal essential medium containing Earle’s salts and supplemented with 2 mM glutamine, 500 µg/mL fungizone, 100 units/mL penicillin, 100 µg/mL streptomycin, and 10% fetal calf serum). An equal volume of either HeV or NiV containing approximately 1 × 10^2^ TCID_50_ was added to each well and the virus–serum mix was incubated for 30 min at 37 °C in a humidified 5% CO_2_ incubator. Then 100 µL of a Vero cell suspension containing 2 × 10^5^ cells/mL was added to each well and the plate was incubated at 37 °C in a humidified 5% CO_2_ incubator. After three days, the wells were observed for signs of viral cytopathic effect (cpe) and the serum titer was determined as the concentration in which cpe was fully neutralized in duplicate wells. All live virus experiments were conducted under strict bio-containment procedures in a BSL-4 laboratory at the Australian Centre for Disease Preparedness.

### 2.10. ACDP HeV DIVA ELISA Used as Reference Test

A Hendra DIVA competitive ELISA was developed at the Australian National Reference laboratory (NRL) for Hendra virus infections at ACDP, and is referred to as a reference test in this study. Briefly, 50 µL of HeV soluble G tetramer antigen, HeV-sG [33] at a concentration of 0.22 µg/mL diluted in PBS was added to columns 1, 3, 5, 7, 9, and 11 of a Nunc Maxisorp ELISA immuno-plate (Thermo Fisher Scientific, Scoresby, VIC, Australia), and 50 µL of yeast-expressed HeV-N antigen (yHeV-N; kindly provided by Kestas Sasnauskas, Vilnius, Lithuania) at a concentration of 0.625 µg/mL diluted in carbonate–bicarbonate buffer (Sigma Aldrich) was added to the alternate columns 2, 4, 6, 8, 10, and 12 on the ELISA plate. Plates were incubated at 37 °C for 1 h with shaking. At the end of the incubation time, 50 µL of casein-blocking buffer (Sigma Aldrich) diluted 1/10 in double-distilled water (ddH_2_O) was added to the plates. Plates were incubated for 30 min at 37 °C with shaking. Plates were washed 6 times with PBS-T using an automatic plate washer (Biotek ELx-405LS, Millennium Science, Mulgrave, VIC, Australia). Then, 50 µL of BB was added to all wells followed by the addition of control sera (high-positive and low-positive Hendra-infected horse sera, and a negative horse serum) to the first two columns of the G and N antigens. Then 10 µL of test samples were added in duplicate to the plate. Plates were incubated at 37 °C for 1 h with shaking. Ten µL of anti-Hendra G mAb 1.2 (White et al., 2005) diluted 1/50 in BB was added to all the sample-containing wells in the Hendra G columns except the blank wells. Also, 10 µL of anti-Nipah N mAb 1g3 (kindly supplied by Kestas Sasnauskas, Institute of Biotechnology, Vilnius, Lithuania) diluted 1/50 in BB was added to all the sample-containing wells in the HeV-N protein columns except the blank wells. Plates were incubated at 37 °C for 30 min with shaking and washed as described above, then 50 µL of HRP-conjugated goat anti-mouse IgG (Jackson Immuno Research Laboratories, Waterford, QLD, Australia) diluted 1/2000 in BB was added to all wells. Plates were incubated at 37 °C for 30 min with shaking. Plates were washed as described above, then 50 µL of TMB substrate (Sigma) was added to all wells. Plates were incubated at RT and the reaction was stopped within 10 min (monitored by the respective monoclonal Ab wells reaching 0.35 OD when read using a 650 nm wavelength), by adding 1 M H_2_SO_4_ (Ajax Finechem, Wollongong, NSW, Australia). Plates were read at 450 nm with a Multiskan FC Microplate plate reader (Thermo Fisher Scientific). Percentage inhibition of the samples were calculated using the OD values obtained relating to their respective Hendra G and N controls using the formula: % inhibition = 100 − (100 × (test serum)/negative control)).

### 2.11. Determination of Analytical Sensitivity (ASe) and Specificity (ASp)

In order to determine the limit of detection (LOD) of the assays or ASe, we analyzed serial dilutions of an infected Australian horse serum in 2.5% skim milk in PBS-T, using dilutions of 1/100 to 1/12,800.

To establish the ASp, 17 serum samples from different species including horses, guinea pigs, pigs, rabbits, and goats [29], containing antibodies raised against different paramyxoviruses (peste des petits ruminants virus, rinderpest virus, canine distemper virus, Newcastle disease virus, parainfluenzavirus Type 1–4, mumps virus, Nariva virus, Tioman virus, Menangle virus, blue eye rubulavirus, Mossman virus) were tested for their reactivity against HeV-G and HeV-N. To allow a species-independent detection of antibodies, we used a biotinylated Protein A/G conjugate (1/500, Pierce) in parallel to the goat anti-horse conjugate in this instance. For the determination of a cut-off value for this modified assay, we tested 280 of the negative German horse sera also in this modified assay using Protein A/G as a conjugate and calculated the cut-off values accordingly.

### 2.12. Evaluation of HeV-N and HeV-G Assays Using Bayesian Latent Class Modelling

Samples were classified as positives and negatives based on the cut-off derived for the FLI assays (HeV-N and HeV-G iELISA) and the assays for detecting N and G antibodies by ACDP. The ACDP assay was used as an ‘imperfect’ reference test.

For estimating the diagnostic specificity (DSp) with 95% confidence and absolute precision of ±2%, assuming sensitivity (Se) of the DIVA assays (HeV-G and HeV-N) could be >99% and specificity (Sp) to be 98%, the minimum required sample size was estimated a priori to be 95 samples for estimating DSe and 188 samples for estimating DSp, using the R package ‘epiR’ [34]. A Bayesian latent class analysis is the OIE-recommended approach [35], where insufficient reference samples are available for such an analysis, making no assumption of the true disease status of the animals from which the samples are derived.

A Bayesian latent class model (BLCM) based on a publication of Branscum and coworkers [36] was fitted with the assumption that both components of the DIVA assay were conditionally dependent—both are antibody-based ELISA tests detecting antibodies against the HeV-G and HeV-N proteins [37,38]; neither test was perfect [39], and the true status of the samples was unknown. The data consisted of the joint results of the FLI and ACDP assays (N and G) obtained from three sample panels listed in Table 1 (panel 2 = negative Australian horses, panel 3 = vaccinated Australian horses; panel 4 = HeV-infected Australian horses) with different prevalences of HeV infection. Because a BLCM that incorporates conditional dependence between tests is not identifiable, prior information on at least two parameters was needed to ensure model identifiability. Given the ACDP DIVA assay is used extensively at the ACDP for detection of horses with possible HeV infections, the DSe and DSp estimates of the ACDP HeV-G and HeV-N assays were used as prior Beta (a,b) distributions for DSe and DSp (based on laboratory data). Beta (a,b) priors were estimated using Betabuster 1.0 (https://betabuster.software.informer.com, accessed on 8 April 2019) in the ‘epiR’ library of the R statistical program [34,40] with assumptions that the DSe was 95% sure to be >0.85 with mode = 0.90, and the DSp estimate was 95% sure to be >0.90 with mode = 0.95. Flat beta (1,1) priors were assumed for the DSe and DSp of the FLI HeV-G and HeV-N assays, given no available prior information. Since both the index test and reference test detected the same antibodies (against HeV-N and HeV-G) we modeled for conditional dependence [38,41].

Panel 4 (Table 1) consisted of HeV-infected horses that were confirmed based on clinical signs, virus isolation, molecular, or serological results (VNT). We assumed a beta prior for disease prevalence with mode = 0.95; 95% sure >0.90. A zero prevalence of the disease in the population was considered for the uninfected horses. Since a beta distribution does not have mass over zero, we modeled true prevalence (π) = π* × τ, assumed probability tau (τ) from a Bernoulli distribution and a probability τ0 if π = 0. With the assumptions that prevalence was 95% sure to be <0.01 with mode = 0.001, the uncertainty about the unknown π* was modeled as π*~beta (aπ, bπ). However, for the vaccinated horses, we calculated the beta priors based on their response to the vaccine (for HeV-G assay) as mode = 0.90 and 95% sure >0.80, and the absence of HeV-N antibodies (due to non-infectious status) as mode = 0.01 and 95% sure <0.05 (for both HeV-N and DIVA assays). Scripts were run using OpenBUGS v3.2.3 [42] with convergence estimates derived using one million iterations of simulation with sampling done every 1000th iteration until the MC error value was <5% of the standard deviation of the node estimate using three assumed chains as initials or three generated initials, and discarding 5000 iterations as burn-in. Convergence was assessed by evaluation of the history, and trace plots and calculation of the Gelman–Rubin statistic diagnostic, which compares the within and between chain variability of the three chains that were run. Posterior medians with 95% probability intervals (PI) corresponding to the 2.5th and 97.5th percentiles of the Monte Carlo sample were used to summarize parameter estimates of DSe, DSp, and apparent prevalence in the three populations.

## 3. Results

### 3.1. Provisional Cut-Off Value Determination for the Indirect DIVA ELISA

**Figure 1:**
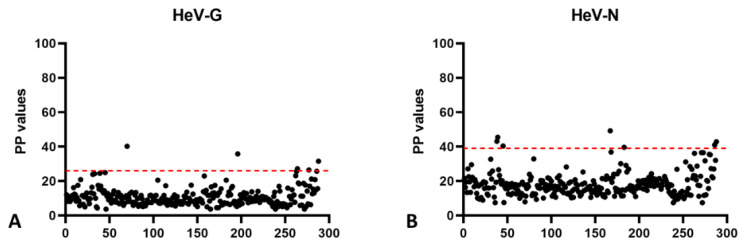
Testing of German negative horse populations and provisional cut-off determination. A total 288 HeV-negative German horse sera were tested in HeV-G and HeV-N ELISA, and the mean PP values plus 3 SD was set as a provisional cut-off. For HeV-G the initial cut-off PP value was determined as 26 (**A**) and for HeV-N as 39 (**B**), as shown by the red dotted lines.

For the determination of a provisional cut-off, 288 German horse samples were tested that had no history of either a HeV vaccination or a possible HeV exposure and were therefore assumed to carry no HeV-specific antibodies in their serum. The mean PP value for HeV-G was 11, and the provisional cut-off was calculated as the mean value + 3 standard deviations (SD), resulting in a cut-off PP value for HeV-G of 26 (Figure 1A). Of the 288 serum samples, 5 samples (numbers 116, 136, 149, 746, and 1099) gave results above the cut-off value, with a maximum PP value of 40, and were therefore selected for further analysis by Western blot analysis, and/or VNT. Likewise, the mean PP value for HeV-N was 18, and the cut-off value (mean PP + 3 SD) was calculated as 39 (Figure 1B). Here, 7 serum samples exceeded this cut-off value (numbers 147, 149, 717, 733, 1065, 1066, and 1072) with a maximum PP value of 49, and were therefore selected for further analysis.

The provisional cut-off values were then confirmed by testing 105 HeV-negative Australian horse sera. The cut-off PP value for HeV-G was calculated as 27 (9.3 plus 3SD) and for HeV N as 33 (10.99 plus 3SD). In this sample set, five sera gave readings above the cut-off values calculated for the Australian samples against HeV G, out of which one sample (number 12-02999-4) also reacted in the ACDP HeV-G ELISA that was used as a reference test. This sample was confirmed negative by VNT. Four other samples gave positive results in the FLI-N-ELISA, none of which were confirmed by the ACDP-N ELISA used as a reference test.

### 3.2. Cut-Off by ROC Analysis and Diagnostic Test Sensitivity (DSe) and Diagnostic Test Specificity (DSp) by Frequentist Approach

**Figure 2:**
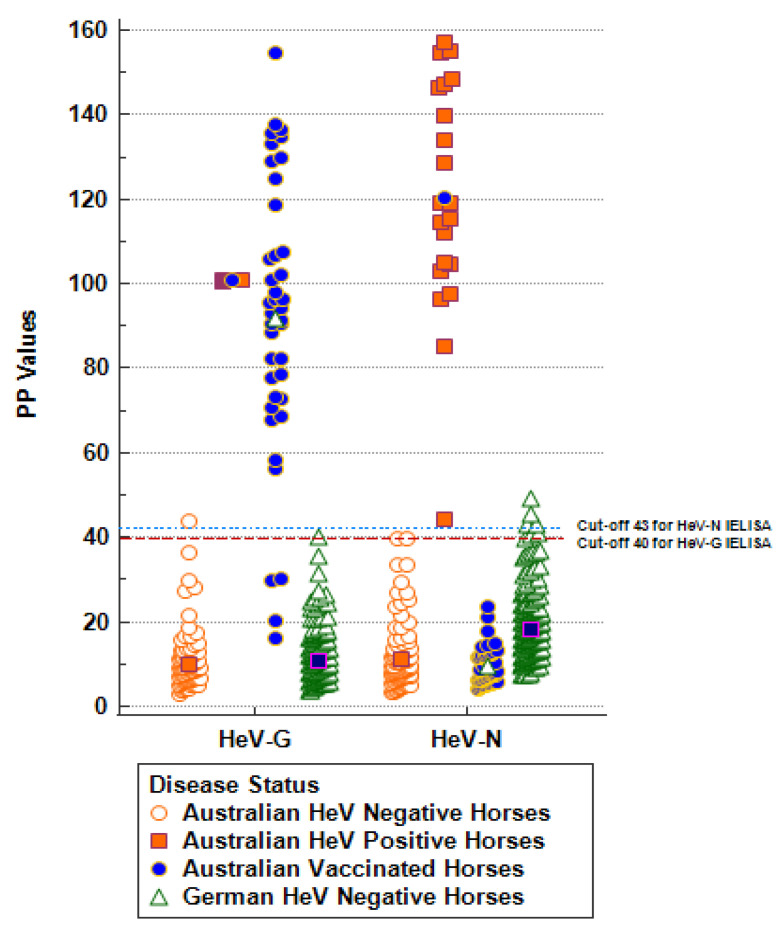
Diagnostic sensitivity (DSe) and specificity (DSp) of HeV-G and HeV-N assays were established using 105 HeV-negative Australian horses, 21 HeV-infected Australian horses, and 40 HeV G-vaccinated Australian horses. The mean values are shown in the middle of the dot plots. Results for 288 HeV-negative German samples and 21 HeV-positive Australian samples were used to derive assay-specific cut-offs.

The performance of the assay was further evaluated by testing serum samples from 105 negative, 40 vaccinated, and 21 infected Australian horses with results initially confirmed using the validated ACDP diagnostic assay (Table 1). While the provisional cut-off values based on PP plus 3SD values were 26 and 39 for HeV-G and HeV-N assays, respectively, based on ROC analysis, the cut-off with the highest combined DSe and DSp for the HeV-G and HeV-N assays were PP values of 40 and 43, respectively (Figure 2). These were therefore set as the final cut-off values for the FLI-DIVA ELISA. Diagnostic samples yielding results between the provisional and the final cut-off are considered questionable and should be re-tested by alternative serological assays (Western blot, IF analysis, or VNT).

All 21 HeV-positive horse sera from infected animals were identified correctly by the HeV-G assay (Figure 2, Table 2). All positive results were confirmed by VNT, with antibody titers determined between 1/16 and 1/4.096. With the exception of one sample (number 11-04096-12) that was positive in the FLI-HeV-G ELISA, but negative in the confirmatory ACDP-HeV-G ELISA and VNT, all Australian negative samples were correctly classified as negative in the HeV-G assay (Figure 2, Table 2). Four out of 40 vaccinated horses did not react in the FLI-HeV-G assay, but were positive in the ACDP-HeV-G assay, while all 40 were correctly identified as non-infected due to non-reactivity in the HeV-N assay (Figure 2, Table 2). The cross-classified counts of joint test results for FLI-HeV-G-ELISA and the reference ACDP-HeV-G ELISA (FLI+, ACDP+; FLI−, ACDP+; FLI−, ACDP−, and FLI−, ACDP−), as well as the panel-specific diagnostic performance, are also shown in Table 2.

Based on ROC analysis using MedCalc^®^, the FLI-HeV-G ELISA tested 104/105 (DSp 99.04%, 95% CI 94.81–99.98%) not-infected Australian horses as negative, 36/40 (DSe 90%, 95% CI 76.34–97.21%) vaccinated horses as negative, and 21/21 (DSe 100%, 95% CI 83.89–100%) infected horses as positive). The FLI-HeV-N ELISA tested 105/105 not-infected (DSp 100%, CI 95% 96.55–100%) and 40/40 (DSp 100%, 95% CI 91.19–100%) vaccinated horses as negative, and 21/21 (DSe 100%, 95% CI 83.89−100%) infected horses as positive (Table 2).

To validate the suitability of this assay for the diagnostic screening of horse sera, we also tested a ring trial panel consisting of blinded samples provided by the Australian NRL. As the aim of the ring trial was the detection of Hendra-positive cases, samples were only tested in the HeV-G ELISA. This set consisted of two negative horse sera, one undiluted vaccinated horse serum, and six samples from two infected horses in pre-dilutions ranging between 1/40 and 1/150, resulting in final dilutions of these sera of 1/4000 and 1/15,000. When the cut-off based on ROC analysis with the highest combined DSe and DSp was applied, two serum dilutions of HeV-infected horses (number 1 pre-diluted 1/150 and number 2 pre-diluted 1/60) were interpreted as negative. However, they were considered positive when the provisional cut-off was applied (Table 3), and these samples would be re-tested using Western blot as diagnostic submissions.

### 3.3. Relative Diagnostic Test Sensitivity (DSe) and Diagnostic Test Specificity (DSp) by BLCM Approach

The results of relative DSe and DSp estimates based on the BLCM are presented in Table 4. The median value of DSe of FLI HeV-G and HeV-N assays with 95% posterior intervals were 0.985 (0.932, 1.000) and 0.902 (0.749, 0.988), respectively, while the median DSp estimates for the FLI HeV-G and HeV-N assays with 95% posterior intervals were 0.994 (0.967, 1.00) and 0.976 (0.939, 0.994) respectively (Supplementary Figure S1). The median DSe and the DSp for the FLI DIVA interpretations (combining the results of HeV-G and HeV-N assays) with 95% posterior intervals were 0.902 (0.749, 0.988) and 0.976 (0.939, 0.994), respectively.

### 3.4. Re-Testing of Reactive Samples by Other Serological Methods

**Figure 3:**
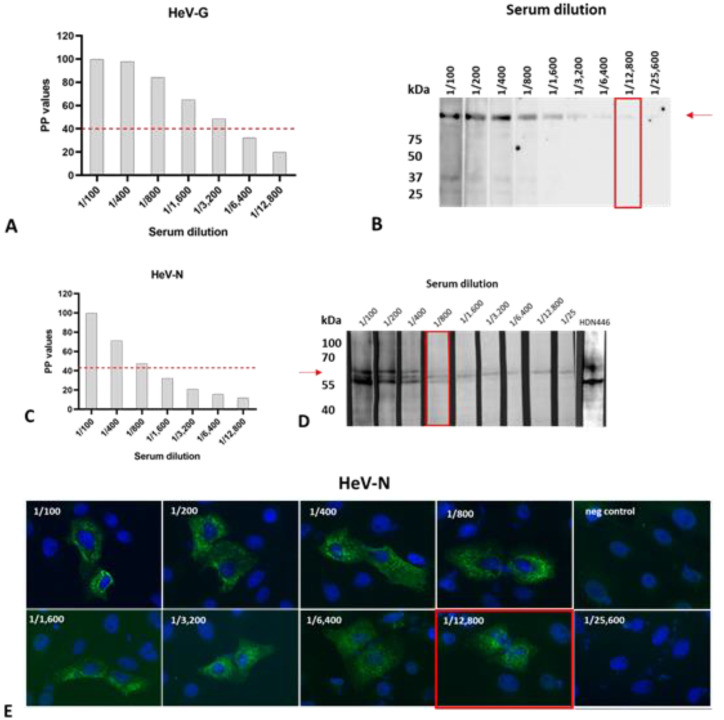
ASe of the ELISA, Western blot, and IF analysis as determined for serial dilutions of a known positive control serum. Detection limits for HeV-G were determined as (**A**) 1/3200 for the HeV-G ELISA using HeV-G expressed in *L. tarentolae* as antigen, and (**B**) as 1/12,800 for the HeV-G Western blot using a commercially available HeV-G expressed in HEK 293T cells as antigen. The detection limits for HeV-N were determined as (**C**) 1/800 for the HeV-N ELISA expressed Sf9 insect cells and (**D**) the HeV-N Western Blot using HeV-N expressed in HEK 293T cells as antigen, and (**E**) as 1/12,800 for IF analysis using HeV-N expressed in Vero76 cells as antigen. Red arrows indicate the expected molecular masses of HeV-G and HeV-N, respectively.

Due to the minimal volume of some of the samples, only four out of five sera that were initially reactive in the HeV-G ELISA and four out of seven sera that were initially reactive in the HeV-N ELISA could be tested further. Unfortunately, the one German serum sample that was positive in the HeV-G and HeV-N assays could not be re-tested in the other assays due to its minimal sample volume. To ensure a sensitivity level of the confirmatory tests that is not inferior to that of the ELISA, we determined the analytical sensitivities of the ELISA, Western blot, and IF, using dilutions of a known positive serum. Here, we used HeV-G and HeV-N antigens that were differently expressed, in order to exclude non-specific reactions related to the expression system. For HeV-G, the detection limit was determined as 1/3200 for the ELISA using HeV-G expressed in *L. tarentolae* as antigen (Figure 3A) and 1/12,800 for the Western blot using a commercially available HeV-G expressed in HEK 293T cells as antigen (Figure 3B). For HeV-N, the detection limit was determined as 1/800 for the ELISA using HeV-N expressed in SF9 insect cells using recombinant baculovirus (Figure 3C), 1/800 for the HeV-N Western Blot using HeV-N expressed in HEK 293T cells as antigen (Figure 3D), and 1/12,800 for IF analysis using HeV-N expressing Vero76 cells (Figure 3E).

**Figure 4:**
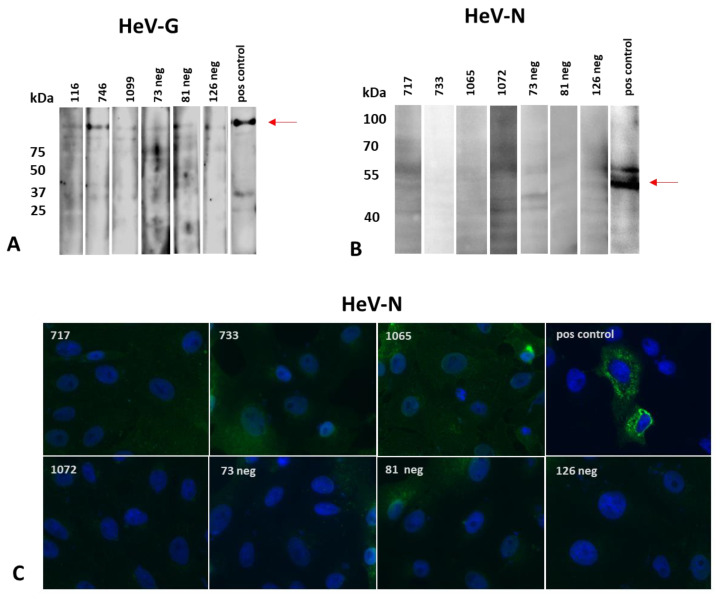
Re-testing of reactive sera by Western blot and IF analysis. (**A**) HeV-G reactive German horse sera numbers 116, 746, and 1099 as well as negative control sera numbers 126, 81, and 73 were tested by Western blot using a commercially available HeV-G expressed in HEK 293T cells as antigen. (**B**) HeV-N reactive German horse sera numbers 717, 733, 1065, and 1072 as well as negative control sera numbers 126, 81, and 73 were tested by Western blot using lysates of HEK 293T cells expressing HeV-N as antigen, (**C**) as well as by IF analysis using Vero76 cells transiently expressing HeV-N. Red arrows indicate expected molecular masses of HeV-G and HeV-N, respectively.

When we tested the available ELISA-reactive samples in the Western blot using a commercially available HeV-G as antigen, one out of four samples (number 746) gave a reaction exceeding that of the known negative samples (numbers 126, 81, 73) that were included in this analysis to allow a better quantification of the background signal that may occur for field serum samples of sub-optimal quality (Figure 4A). None of the analyzed samples reacted in the Western blot using HeV-N expressed in HEK 293T cells as antigen (Figure 4B), or in the IF analysis using HeV-N expressed in Vero E6 cells (Figure 4C). Sample 746 was further analyzed by VNT, with a negative result.

### 3.5. Determination of Analytical Sensitivity (ASe) and Specificity (ASp)

To determine and compare the ASe of the DIVA ELISA, Western blot, and IF analysis, we analyzed serial dilutions of a known positive horse serum in all three assays (Figure 3). In order to allow the species-independent analysis of serum samples to determine the ASe, we used Protein A/G instead of the horse-specific conjugate. We therefore re-tested 280 of the German negative horse sera in the modified HeV-G and HeV-N assays using Protein A/G as a conjugate, and determined cut-off PP values of 26 and 55, respectively. The detection limit was 1/3200 for the detection of HeV-G-specific antibodies using both conjugates, but was inferior when using Protein A/G for the detection of specific antibodies against HeV-N (1/400 versus 1/800 when using the horse-specific conjugate) (Supplementary Figure S2).

Then, to analyze the ASp, 17 serum samples from different species including horses, pigs, guinea pigs, rabbits, and goats, containing antibodies raised against different paramyxovirus antigens were tested using the modified DIVA ELISA using Protein A/G as a conjugate (Table 1, panel 5). Testing yielded negative results for all sera with HeV-G as antigen, and for 15 of the sera with HeV-N as antigen, while one horse anti-parainfluenza Type 3 virus and one horse anti-mumps-virus serum was positive when tested with the anti-horse conjugate and negative when tested with Protein A/G, and one rabbit anti-Mossman-virus serum was positive when developed with Protein A/G (Table 5), showing a satisfying ASp of both assays.

## 4. Discussion

We have developed and evaluated a HeV DIVA ELISA assay that not only allows the detection of positive horse sera, but also discrimination between HeV-vaccinated and -infected horses. DIVA approaches are also considered crucial for an effective control of other animal diseases where vaccines are available, such as classical swine fever [43], equine influenza [44], avian influenza [45,46], foot and mouth disease [47], and Aujeszky’s disease [48]. This discrimination is indispensable for an effective and economic disease control, as it allows the safe exclusion of vaccinated animals from culling schemes. Thus, the availability of serum samples from confirmed negative, HeV-G-vaccinated, and HeV-infected horses was as a prerequisite for the evaluation of our assay. Since the number of available serum samples from HeV-vaccinated or -infected horses was limited, and the evaluation therefore had to be performed with relatively small sample panels for these populations, we performed a Bayesian latent class analysis and a reference standard approach for the evaluation of this assay. This assay will be mostly used for the confirmation of freedom from HeV infection in serum samples from horses originating from Europe and possibly North America. Given the higher diagnostic sensitivity of the HeV-G ELISA, this assay would be preferable if the presence of serum samples from vaccinated animals could be excluded, and a differentiation between infected and vaccinated animals was not needed. Testing negative horse populations from Germany and Australia resulted in similar provisional cut-off PP values, i.e., 26 and 39 for German horse samples, and 27 and 33 for Australian horse samples for the HeV-G and HeV-N ELISAs, respectively. This indicates that this assay is suitable for the testing of horse samples from Europe and Australia and possibly other geographical regions. From our own previous study using the same HeV-G antigen, we know that this assay is not only suitable for the detection of HeV-specific antibodies, but would also be suitable to detect a possible NiV infection [32].

The reported evaluation study demonstrated a very good performance of this assay in detecting HeV-positive sera. This was supported by its performance in the ring trial organized by the Australian NRL, where all positive samples were identified as positive or questionable (two diluted samples), which in the case of diagnostic submissions would need to be re-tested by Western Blot or IF analysis. During the determination of the cut-off values using negative serum samples, we did identify individual samples that were reactive either with the HeV-G or with the HeV-N antigen. These were then re-tested by Western blot and IF analysis using differently expressed HeV G or N antigens, to avoid reactions to HeV-unrelated antigens originating from the expression system leading to false-positive results. Samples that gave questionable results in these assays were then re-tested using the VNT (which does not distinguish between sera from vaccinated and infected horses). A recent study has confirmed that the application of three doses (two primary vaccinations 3–4 weeks apart plus a third vaccination after 6 months) resulted in the detection of neutralizing antibodies in 100% of the horses [49], underlining the suitability of the VNT to confirm positive ELISA results in fully vaccinated horses. Prior to using these assays for confirmation purposes, we established their comparative analytical sensitivities, to ensure that these were at least equal to that of the ELISA. Interestingly, out of the 399 negative samples that were tested altogether, we only identified one sample (German horse number 149) that gave a positive result for both HeV-G and HeV-N. Unfortunately, this sample could not be re-tested using the other assays due to its minimal volume, but since this animal did not have a history of travelling to any country where HeV or NiV infections in horses have ever been reported, it must be assumed that these were indeed false positive reactions, possibly due to the poor sample quality, or due to a cross-reaction with another unrelated antigen. All other false-positive results concerned only one antigen, and were not confirmed by any of the other applied tests.

As shown by the analysis of serial dilutions of a known positive sample, the analytical sensitivity of the HeV-N-based ELISA is inferior to that of the HeV-G-based ELISA (threshold of 1/800 and 1/3200). Thus, a sample that is positive in the HeV-G ELISA and negative in the HeV-N ELISA should be re-tested using IF analysis on cells expressing HeV-N for confirmation of the HeV-N negative result, as we determined the threshold of detection of this method to be a 1/12,800 dilution of the same positive sample.

To be able to determine the analytical specificity of our assay on a panel of paramyxovirus-positive serum samples from different species, we needed to apply a slightly modified protocol, using Protein A/G instead of a horse-specific conjugate. Testing of 280 German negative horse sera resulted in a cut-off PP value of 26 for HeV-G, which is almost identical to that determined using the horse-specific conjugate. Also, analysis of a serial dilution of the positive control serum revealed a detection threshold of 1/3200 for the HeV-G ELISA (the same as when using the horse-specific conjugate) and of 1/400 for the detection of HeV-N-specific antibodies (1/800 when using the horse-specific conjugate). This indicates that the modified ELISA protocol will be suitable for the testing of serum samples derived from other species, such as pigs, dogs, ruminants, or bats, which have been reported to be susceptible to infections with HeV or other closely related henipaviruses [4,5,50,51,52]. Given the high level of cross-reactivity between HeV, NiV and the so-far-known African henipaviruses, this assay could then also be applied to screen for antibodies against these agents [26,27,53,54]. However, further validation efforts would be required before such assays could be applied for diagnostic purposes. In the past, serological analysis of different species for the presence of Henipavirus-specific antibodies has mostly been done using microsphere-based assays [26,53,54]. Although this approach offers the enormous advantage of being able to analyze samples in a multiplex analysis for antibodies against several disease agents, the expensive microsphere readers required are not available in many institutions, while ELISA readers are very widely distributed. Moreover, if used as a single assay, the microsphere-based assay is distinctly more expensive than the ELISA.

From the vaccinated sample panel, 4 out of 40 tested horses were not detected as positive in the HeV-G assay, but were positive in the reference assays established at the Australian NRL. All 40 were correctly identified as non-infected due to non-reactivity in the HeV-N assay. Since the analyses of these samples with both methods were performed up to four years apart, a degradation of antibodies in the sample cannot be fully excluded.

Analysis of data obtained with the BLCM resulted in high estimates for DSe and DSp for the HeV-G assay, 98% and 99%, and for the HeV-N with 95% and 98%, respectively. When we applied a frequentist approach, ROC analysis resulted in slightly higher estimates; the HeV-G assay had a DSe of 100% and a DSp of 99% (cut-off at 40 PP value), and the HeV-N had a DSe of 100% and a DSp of 100% (cut-off at 43 PP value).

Performing ROC analysis on samples from negative, vaccinated and infected populations tested in the HV-G ELISA revealed only one false positive sample in the Australian negative horse panel and four false negatives in the vaccinated horse panel when compared to the ACDP HeV-G ELISA. Overall, the FLI HeV DIVA ELISA shows good discrimination between negative, vaccinated, and infected horses. In rare circumstances PP values as high as 40 and 49 were produced by the HeV-G and HeV-N ELISA respectively in the negative population of horses from Germany. For the HeV-G and HeV-N ELISA a cut-off PP value around 40 and 43 respectively will favor a high DSe and DSp. It should be further explored whether the ratio between HeV-G and HeV-N PP values of a test result can be used to increase the certainty of the combined results interpretation. For example, using the mean PP values from German negative horse samples, and the Australian vaccinated and infected horse samples from the HeV-G and HeV-N ELISA results in the following ratios, i.e., negative: G 11/N 18 = 0.61; vaccinated: G 93/N 10 = 9; infected: G 101/N 122 = 0.83. Ratios of G/N around 8 could be used to corroborate individual results for G and N from a potentially vaccinated horse and ratios around 1 or less would indicate a negative or infected horse depending on the individual results for G and N. More data needs to be generated to confirm this hypothesis.

In summary, we present a DIVA assay for the detection and discrimination of serum samples from HeV-infected and HeV-vaccinated horses, which can be applied on serum samples from different geographical regions. A modified assay protocol is suitable for the analysis of samples originating from different species.

## Figures and Tables

**Table 1:** Serum panels used for development and evaluation of the FLI HeV DIVA ELISA using HeV-G and HeV-N proteins.

Panel	Purpose, Determination of	Source of Serum	Assays Performed
1	Cut-off DSp	**German negative horses (*n* = 288)**; These samples had been submitted from different clinics to the National Reference Laboratory for West Nile Virus (WNV) between 2009 and 2012 for WNV screening. None of the horses had a history of travelling to Australia or being vaccinated against HeV, and these samples were therefore considered HeV-negative	Preliminary Cut-off determination ROC curve analysis FLI HeV DIVA ELISA, ACDP HeV DIVA ELISA, HeV VNT
2	Cut-off DSp	**Australian negative horses (*n* = 105)**	Cut-off determination ROC curve analysis BLCM FLI HeV DIVA ELISA, ACDP HeV DIVA ELISA, HeV VNT
3	Cut-off DSe, DSp	**Australian vaccinated horses (*n* = 40)**; diagnostic field samples	FLI HeV DIVA ELISA, ACDP HeV DIVA ELISA BLCM
4	Cut-off DSe ASe	**Australian HeV-positive samples (*n* = 21)** from outbreak episodes (QLD) and follow-up testing	Cut-off determination and ROC curve analysis BLCM FLI HeV DIVA ELISA, ACDP HeV DIVA ELISA, HeV VNT
5	ASp	**Serum samples originating from different species (*n* = 17)** including horses, guinea pigs, pigs, rabbits, and goats, containing antibodies against different paramyxoviruses (peste des petits ruminants virus, rinderpest virus, canine distemper virus, Newcastle disease virus, parainfluenzavirus type 1–4, mumps virus, Nariva virus, Tioman virus, Menangle virus, blue eye rubulavirus, Mossman virus)	FLI HeV DIVA ELISA

FLI = Friedrich Loeffler Institut; ACDP = Australian Centre for Disease Preparedness; QLD = Queensland; ASe = analytical sensitivity; ASp = analytical specificity; DSe = diagnostic sensitivity; DSp = diagnostic specificity; BLCM = Bayesian latent class model.

**Table 2:** Diagnostic performance of HeV G and HeV N at cut-off PP values of 40 and 43, respectively (frequentist approach). The number of horses positive in each population is given in brackets.

Population		HeV-G Assay			HeV-N Assay	
	Cross-Classified Counts *	Diagnostic Sensitivity (DSe)	Diagnostic Specificity (DSp)	Cross-Classified Counts *	Diagnostic Sensitivity (DSe)	Diagnostic Specificity (DSp)
German negative horse sera	n.a.	n.a.	99.04%	n.a.	n.a.	99.30%
			(284/288)			(286/288)
Australian negative horse sera	0, 1, 0, 104	n.a.	99.04%	0, 0, 0, 105	n.a.	100%
			(104/105)			(105/105)
Australian vaccinated horse sera	36, 0, 4, 0	90.00%	n.a.	0, 0, 0, 40	n.a.	100%
		(36/40)				(40/40)
Australian HeV-infected horse sera	21, 0, 0, 0	100%	n.a.	21, 0, 0, 0	100%	n.a.
		(21/21)			(21/21)	

* Cross-classified counts of joint test results for FLI-HeV-G-ELISA and the reference ACDP-HeV-G ELISA (FLI+, ACDP+; FLI−, ACDP−; FLI+, ACDP−, and FLI−, ACDP−); n.a. = not applicable.

**Table 3:** Test results of ring trial samples in HeV-G ELISA. When applying the cut-off PP values based on ROC analysis with the highest combined DSe and DSp of 40, two dilutions of positive serum samples were identified as questionable and would be repeated by Western Blot or IF analysis.

Sample ID	Sample Information	PP	Result	Interpretation
neg control		8.55	neg	
pos control		100.00	pos	
RTS 1	infected horse number 1; 1/100	47.72	pos	correct
RTS 2	vaccinated horse	76.57	pos	correct
RTS 3	infected horse number 1; 1/150	35.28	neg	questionable
RTS 4	infected horse number 2; 1/60	43.59	pos	correct
RTS 5	negative horse	8.21	neg	correct
RTS 6	infected horse number 2; 1/40	42.36	pos	correct
RTS 7	infected horse number 2; 1/60	31.43	neg	questionable
RTS 8	infected horse number 2; 1/40	59.40	pos	correct
RTS 9	negative horse	10.25	pos	correct

**Table 4:** Estimates of relative diagnostic sensitivity (DSe) and specificity (DSp), population prevalence and correlation coefficient for negatives and positive results, based on the BLCM for HeV-G, HeV-N, and the DIVA interpretations based on the two assays. (Pr—prevalence; PM—posterior median; 95% PI—95% posterior intervals).

Estimates	HeV-G Assay		HeV-N Assay		DIVA Assay	
	PM	95% PI	PM	95% PI	PM	95% PI
DSe_ACDP_	0.919	0.872, 0.953	0.908	0.852, 0.950	0.910	0.854, 0.948
DSp_ACDP_	0.942	0.903, 0.968	0.954	0.923, 0.976	0.952	0.915, 0.975
DSe_FLI_	0.985	0.932, 1.000	0.953	0.826, 0.998	0.941	0.810, 0.997
DSp_FLI_	0.994	0.967, 1.000	0.975	0.938, 0.994	0.993	0.965, 1.000
Pr_Australian Infected horses_	0.953	0.908, 0.981	0.953	0.907, 0.981	0.954	0.912, 0.980
Pr_Australian Negative horses_	0.002	0, 0.009	0.002	0, 0.009	0.030	0.013, 0.060
Pr_Australian Vaccinated horses_	0.939	0.877, 0.977	0.014	0.002, 0.046	0.057	0.022, 0.113
Correlation coefficient negative (rhoDc)	0.185	−0.016, 0.678	0.325	−0.027, 0.872	0.370	−0.027, 0.865
Correlation coefficient positive (rhoD)	0.124	−0.012, 0.475	0.094	−0.030, 0.442	0.142	0, 0.554

**Table 5:** PP results of analytical specificity (ASp) for HeV-G and HeV-N assays with 17 sera from different species containing antibodies against different paramyxoviruses. Positive results are printed in red, including one horse anti-parainfluenza Type 3 virus and one horse anti-mumps-virus serum when developed with the anti-horse conjugate, and one rabbit anti-Mossman-virus serum when developed with Protein A/G. n.a.—not available.

Sera Used for Analytical Specificity (ASp)	Anti-Horse-HRP		Protein A/G-HRP	
	HeV G PP Value	HeV N PP Value	HeV G PP Value	HeV N PP Value
No serum control (NSC)	1.81	40.44	1.50	7.80
Negative horse serum	1.05	34.85	5.50	6.50
HeV-infected horse	100.00	100.00	100.00	100.00
Caprine anti-PPR 1.4.78	n.a.	n.a.	2.60	7.90
Rabbit anti-rinderpest 5.6.78	n.a.	n.a.	20.60	10.80
Horse anti-canine-distemper-virus 8807-25-0205	3.23	21.98	16.40	11.60
Rabbit anti-NDV V4 8604-28-4425	n.a.	n.a.	8.50	12.90
Horse anti-parainfluenza type 1 (Sendai) 4.17.68	2.67	25.36	3.50	11.90
Horse anti-parainfluenza type 2 (SV-5) 11.14.66	1.99	20.94	20.10	10.90
Horse anti-parainfluenza type 3 (C-243) 1.5.64	3.29	53.97	1.50	6.70
Guinea pig anti-parainfluenza type 1 (C-39) Nov 1964	n.a.	n.a.	2.70	7.80
Guinea pig anti-parainfluenza type 4A (M-25)	n.a.	n.a.	1.60	6.40
Guinea pig anti-parainfluenza type 4B (19503)	n.a.	n.a.	1.60	6.10
Horse anti-mumps Enders strain 8.9.66	6.53	43.95	1.50	6.90
Rabbit anti-Nariva number 1 22/11/2001	n.a.	n.a.	1.60	6.10
Pig anti-Tioman-virus P298 26/09/05	n.a.	n.a.	1.80	51.80
Rabbit anti-Menangle-virus number 4	n.a.	n.a.	3.50	6.40
Pig anti-Menangle-virus number 1 Sept 1999	n.a.	n.a.	3.90	6.70
Pig anti-blue-eye-rubulavirus 9/9/80	n.a.	n.a.	7.90	6.70
Rabbit anti-Mossman-virus 27/07/2000	n.a.	n.a.	1.90	57.1
cut-off	40	43	26.1	55.4

## Data Availability

Data is contained within the article or supplementary material.

## Supplementary Materials

The following are available online at https://www.mdpi.com/article/10.3390/microorganisms10061095/s1, Figure S1: Prior and posterior diagnostic estimates for relative diagnostic sensitivity, diagnostic sensitivity for HeV-G and HeV-N assays using BLCM approach, Figure S2: Analytical Sensitivity of HeV-G and HeV-N FLI-ELISA using Protein A/G as conjugate.
