# Supplementary material for: Serological Hendra Virus Diagnostics Using an Indirect ELISA-Based DIVA Approach with Recombinant Hendra G and N Proteins
Source: Microorganisms. 2022 May 25;10(6):1095. doi: 10.3390/microorganisms10061095 (PMC9230382; doi:10.3390/microorganisms10061095)

Supplementary Figure S1: Prior and posterior diagnostic estimates for relative diagnostic sensitivity, diagnostic sensitivity for HeV-G and HeV-N assays using BLCM approach.

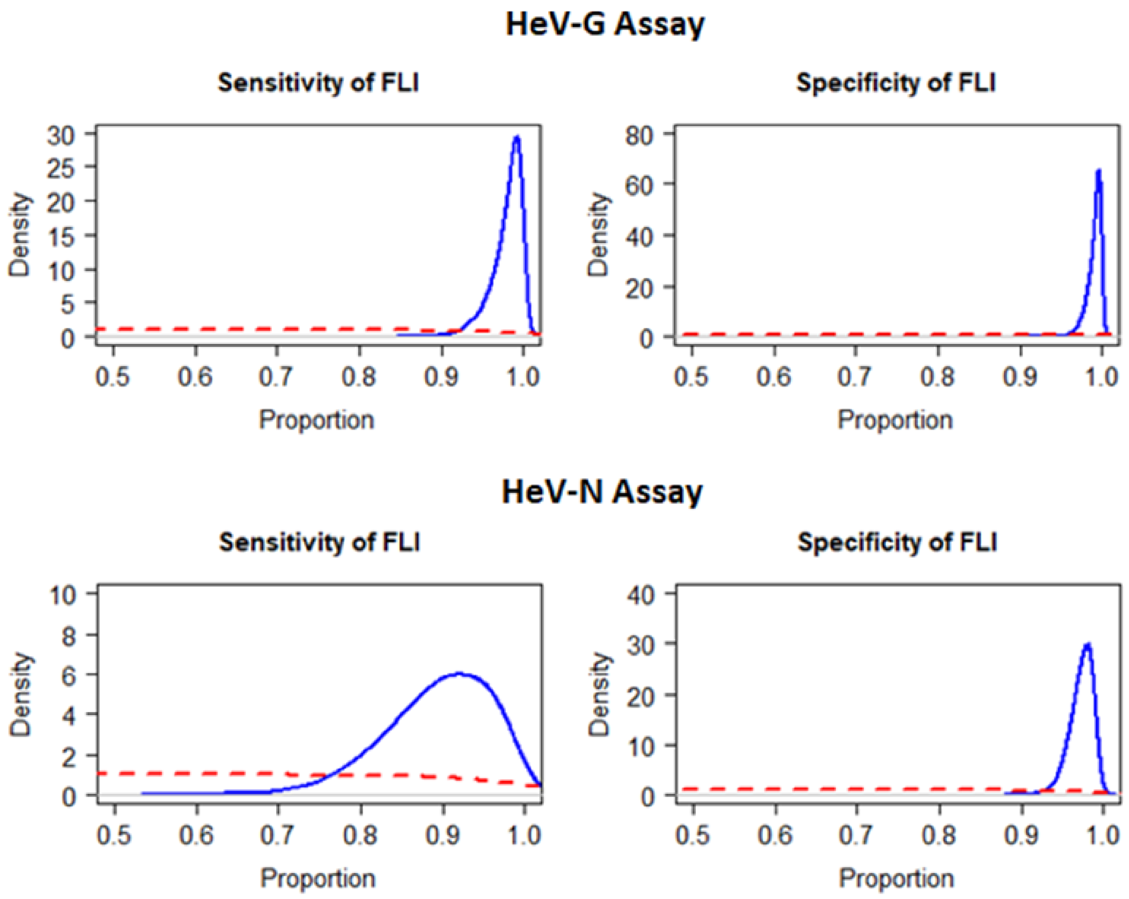

Supplementary Figure S2: Analytical Sensitivity of HeV-G and HeV-N FLI-ELISA using Protein A/G as conjugate.

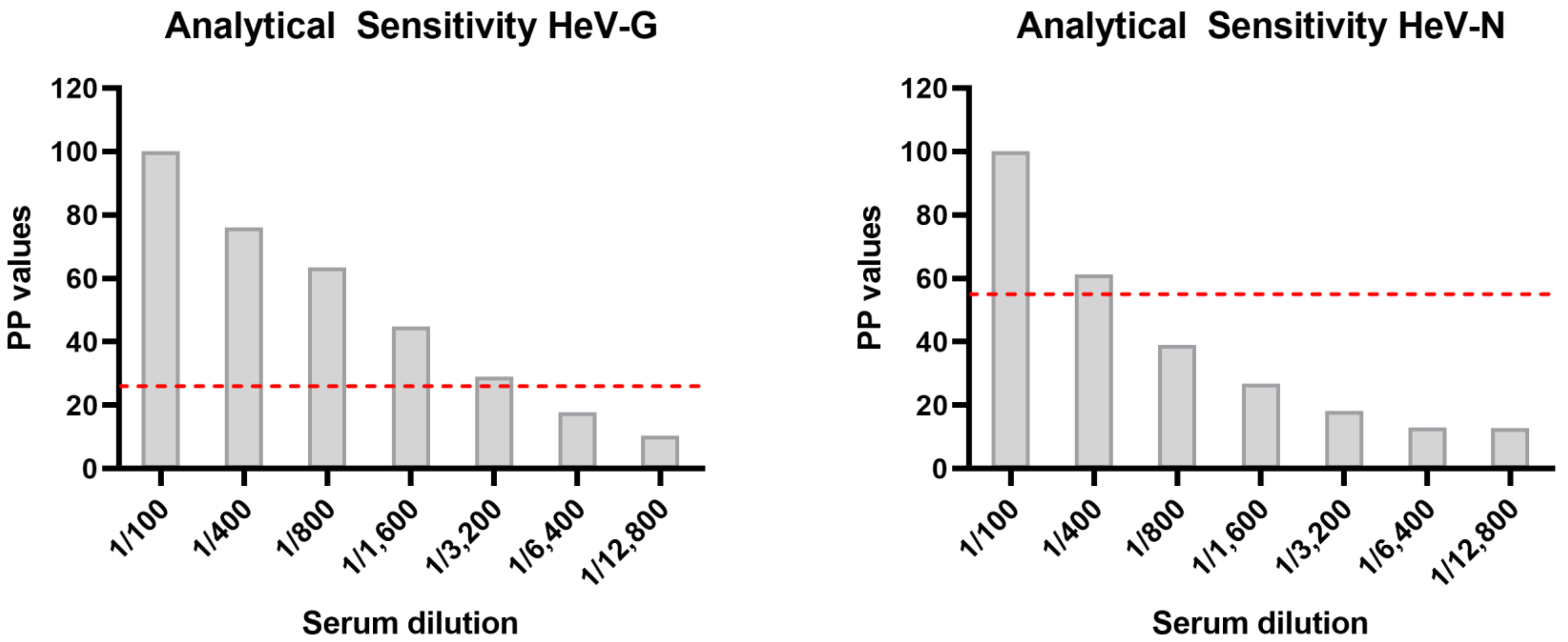

Supplement: Supplementary file 1 [file microorganisms-10-01095-s001.zip › microorganisms-1711915-supplementary.pdf]
